# Total Polyphenols Content, Antioxidant and Antimicrobial Activities of Leaves of *Solanum elaeagnifolium* Cav. from Morocco

**DOI:** 10.3390/molecules27134322

**Published:** 2022-07-05

**Authors:** Mohammed Bouslamti, Azeddine El Barnossi, Mohammed Kara, Badriyah S. Alotaibi, Omkulthom Al Kamaly, Amine Assouguem, Badiaa Lyoussi, Ahmed Samir Benjelloun

**Affiliations:** 1 Laboratories of Natural Substances, Pharmacology, Environment, Modeling, Health and Quality of Life (SNAMOPEQ), Faculty of Sciences, Sidi Mohamed Ben Abdellah University, Fez 30000, Morocco; mohammed.bouslamti@usmba.ac.ma (M.B.); badiaa.lyoussi@usmba.ac.ma (B.L.); ahmed.samir.benjelloun@usmba.ac.ma (A.S.B.); 2 Laboratory of Biotechnology, Environment, Agri-Food and Health, Faculty of Sciences Dhar El Mahraz, Sidi Mohammed Ben Abdellah University, Fez 30050, Morocco; azeddine.elbarnosi@usmba.ac.ma; 3 Laboratory of Biotechnology, Conservation and Valorisation of Natural Resources (LBCVNR), Department of Biology, Faculty of Science Dhar El Mahraz, Sidi Mohamed Ben Abdellah University, Fez 30000, Morocco; 4 Department of Pharmaceutical Sciences, College of Pharmacy, Princess Nourah bint Abdulrahman University, P.O. Box 84428, Riyadh 11671, Saudi Arabia; bsalotaibi@pnu.edu.sa (B.S.A.); omalkmali@pnu.edu.sa (O.A.K.); 5 Laboratory of Functional Ecology and Environment, Faculty of Sciences and Technology, Sidi Mohamed Ben Abdellah University, Imouzzer Street, Fez 30000, Morocco; assougam@gmail.com

**Keywords:** *Solanum elaeagnifolium*, polyphenols, flavonoids, antimicrobial activity, antioxidant activity

## Abstract

*Solanum elaeagnifolium* is among the invasive plants of Morocco; studies on its chemical composition and biological activities are few in number in Morocco. *S. elaeagnifolium* has shown molluscicidal and nematicidal and cancer-inhibitory effects, anti-inflammatory, analgesic activity, and antibacterial activity. The objective of this research is to improve this plant and assess its antibacterial and antioxidant properties as well as its total polyphenolic content (TPC) and total flavonoid content (TFC). The Folin-Ciocalteu method and the aluminium-trichloride method were used to determine TPC and TFC in hydro-ethanolic (HEE) and hydro-acetonic (HAE) leaf extract. Three assays were performed to determine the antioxidant activity: the DPPH test (radical 2,2’-diphenyl-1-picrylhydrazyl), the FRAP test (Ferric Reducing Antioxidant Power), and the TAC test. Disk diffusion and microdilution were used to test antibacterial activity against four pathogenic bacteria and *Candida albicans*. The hydro-ethanolic extract 2.54 ± 0.4 mg EAG/g has a greater polyphenol concentration than the hydro-acetonic extract 1.58 ± 0.03 mg EAG/g. Although the flavonoid content of the hydro-acetonic extract (0.067 ± 0.001 mg EQ/g) is larger than that of the hydro-ethanolic extract (0.012 ± 0.001 mg EQ/g), the flavonoid content of the hydro-ethanolic extract (0.012 ± 0.001 mg EQ/g). The DPPH values were IC-50 = 0.081 ± 0.004 mg/mL for hydro-ethanoic extract and 0.198 ± 0.019 mg/mL for hydro-acetonic extract, both extracts superior to BHT (0.122 ± 0.021 g/mL). While the FRAP assay showed a low iron-reducing power values for both extracts compared to BHT), the overall antioxidant activity of the two extracts was found to be considerable. The overall antioxidant activity of the hydro-ethanolic extract was 8.95 ± 0.42 mg EAA/g, whereas the total antioxidant activity of the hydro-acetonic extract was 6.44 ± 0.61 mg EAA/g. In comparison with the antibiotic Erythromycin, HAE and HEE from *S. elaeagnifolium* leaves demonstrated significant antibacterial action. HAE had the best inhibitory efficacy against *Bacillus subtilis* DSM 6333, with an inhibition diameter of 10.5 ± 0.50 mm and a MIC of 7.5 ± 0.00 mg/mL, as well as against *Proteus mirabilis* ATCC 29906, with an inhibitory diameter of 8.25 ± 0.75 mm and a MIC of 15 ± 0.00 mg/mL.

## 1. Introduction

For thousands of years throughout the history of humanity, Traditional treatments have included the use of medicinal herbs, and they continue to form the foundation of ethnomedicine for many centuries from around world [1,2]. The therapeutic using plants for the therapy of human disorders is very old and evolved with the history of humanity [3]. In fact, there are around 500,000 plant species on the planet, 80,000 of which have therapeutic characteristics [4].

Among the molecules found in medicinal plants are polyphenols. Polyphenols, also known as phenolic chemicals, are secondary metabolites that are present in all fruits and vegetables as well as other parts of the flowering plant. Every parts of the plant contain these chemicals but with a quantitative distribution that varies between different tissues. Several polyphenols have been identified [5]. Phenolic acids, for example, are simple molecules, whereas tannins, for example, are highly polymerised compounds [6].

The medicinal and therapeutic virtues of plants are due to their richness in secondary metabolites, known as active principles, that act directly on the body [7,8]. Recent research on secondary metabolites is very advanced, especially in the fields of herbal medicine and food hygiene, because of their various biological properties: antioxidant, antimicrobial, hypoglycemic, anti-inflammatory, etc. [9]. These metabolites may also have advantageous physiological effects in the cancer prevention and many other chronic disorders, including cardiovascular ailments [10].

For a very long time, foods have been preserved from oxidation by the addition of antioxidants to them [11]. Free radicals are highly reactive chemicals generated when oxygen interacts with particular molecules. They are chemical species with an odd or unpaired electron. They are neutral, short-lived, unstable, and very reactive when it comes to pairing with the odd electron and achieving a stable structure. Once produced, these extremely reactive radicals can start a chain reaction in the body, causing healthy cells to lose their structure and function. Cells could malfunction or perish in this situation [12].

*Solanum elaeagnifolium* is adapted to many types of habitat in various regions around the world. It is a thermophilic species that thrives in warm, temperate climates and can be found at an altitude of 1200 m. Its annual fluviometry ranges from 250 to 600 mm. It can accommodate various soil textures [13].

The Solanaceae family includes *S. elaeagnifolium*. This species is common in America and is spreading to many other countries, including Spain and the Maghreb [14]. In the Family solanaceae, the *Solanum* genus is the richest, including around 2000 species. The most common foods derived from this genus are the tomato (*Solanum lycopersicum*), the potato (*Solanum tuberosum*), the eggplant (*Solanum melongena*), *Solanum nigrum*, and *Solanum elaeagnifolium* [15]. These plants’ positive effects on human health have been linked to the quantity of phenols, alkaloids, saponins, terpenes, flavonoids, coumarins, and carotenoids that they contain [16]. Certain substances have been shown to have anticancer, antioxidant, antihypertensive, antidepressant, anti-inflammatory, hypoglycemic, hypolipidemic, hepatoprotective, anti-obesogenic, and antidiabetic properties [17]. Extracts of *S. elaeagnifolium*, have shown molluscicidal and nematicidal activities as well as cancer-inhibitory activity [18,19,20]. The fruits of *S. elaeagnifolium* showed hepatoprotective [21], anti-inflammatory, and analgesic activities [22]. Extracts from the stem and leaves have exhibited antimicrobial effect against pathogenic strains [23].

Our research’s goal is to screen the leaves of *S. elaeagnifolium* for phytochemicals and assess their antioxidant and antibacterial properties. 

## 2. Materials and Methods

### 2.1. Plants Material 

**Figure 1:**
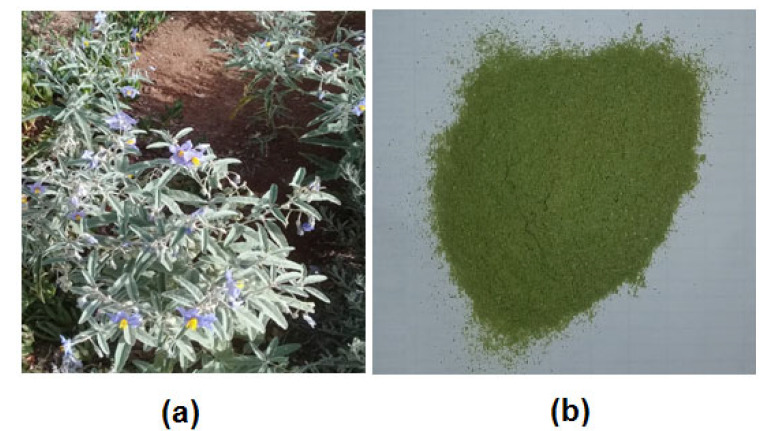
Plant of *S. elaeagnifolium*—(**a**); powder of *S. elaeagnifolium*—(**b**).

End of November 2021 saw the collection of *S. elaeagnifolium* leaves in the Moroccan city of Fez (34°04′04.2 N, 5°01′26.4 W). Professor Bari Amina from the Department of Life Sciences at the University of Sidi Mohamed Ben Abdellah conducted the identification (voucher number: E17/14054). The material was processed via an electric mill into a powder form, and then dried in the shade beside a well (Figure 1). The powder was then preserved in squeaky-clean plastic boxes.

### 2.2. Preparation of Extracts by Maceration

The leaves of *S. elaeagnifolium* were used to generate the hydro-ethanolic extract (HEE) and hydro-acetonic extract (HAE); 10 g of the powdered leaves were combined with 70 mL of ethanol and 30 mL of distilled water to obtain the HEE, and 70 mL of acetone and 30 mL of distilled water to obtain the HAE [24]. Before filtering the extracts, they were macerated for 72 h. A rotary evaporator was used to evaporate the solvent. The extracts were kept at 4 °C until they were used. According to the following formula, the extraction yield has been calculated as a percentage of the weight of the plant powder used:

Y (%) = (EW/WP) × 100

Y: yield of extract in %; EW: extract weight obtained; WP: weight of the powder.

### 2.3. Phytochemical Study

#### 2.3.1. Phytochemical Screening

The two leaf extracts of *S. elaeagnifolium* were subjected to qualitative reactions as part of the phytochemical research to identify the various families of chemicals present. As a result, we conducted a phytochemical screening to search for the following secondary metabolites: tannins, flavonoids, steroids, alkaloids, polyphenols, and saponins are among some of the compounds from plants [25].

#### 2.3.2. Total Polyphenols

According to Li et al. [26], the Folin-Ciocalteu technique is employed to estimate the total polyphenol content. 100 µL of each extract were joined with 500 µL of the Folin-Ciocalteu reagent (10 %). After 4 min, 400 µL of a 7.5% sodium carbonate (Na_2_CO_3_) solution was added. Before estimating the absorbance at 760 nm, the combination was prepared by reacting at room temperature (25 + 1 °C) for two hours. Milligrams of gallic acid equivalents (mg GAE/g extract) were used to express the results.

#### 2.3.3. Flavonoids Content

Kosalec et al. [27] used the aluminium-chloride colourimetric technique to measure the flavonoid content of extracts. We prepared 1 mL of extract and 1 mL of 2% aluminium chloride in methanol. The absorbance at 430 nm has been measured after 30 min of incubation, and quercetin equivalents per gram of extract (mg QE/g extract) are used to represent the estimated flavonoid content.

### 2.4. Antioxidant Activity

#### 2.4.1. Free Radical Scavenging Activity in the DPPH

The antioxidant activity of the two *S. elaeagnifolium* extracts studied was estimated in terms of hydrogen donor or free radicals, using the stable radical 2,2’-diphenyl-1 picrylhydrazyl (DPPH) as a reagent at a concentration of 4 mg/100 mL. When this radical encounters a proton donor, such as an antioxidant, it changes colour from purple to yellow with a decrease in absorbance.

Briefly, 50 µL of various concentrations of the produced samples were added to 850 µL of DPPH solution in ten test tubes. Absorbance measurements were taken at 517 nm after 20 min in the dark at room temperature (25 ± 1 °C). As a standard solution, quercetin was used (positive control). The DPPH radical’s percentage of inhibition was calculated by using the formula:

Inhibition% = (AB—AE/AB) × 100

where, AB is the uptake of the control (-); and AE is the uptake of the extract [28].

#### 2.4.2. Reductive Power Test (FRAP)

The two *S. elaeagnifolium* extracts under investigation had their reduction capacity assessed using the procedure outlined by Zovko Konci et al. [29]. We mixed 200 µL of the extract with known concentration with 500 µL of phosphate buffer (0.2 M, pH 6.6) and 500 µL of 1% potassium ferricyanide K_3_[Fe(CN)_6_]. The final solution was stirred for 20 min at 50 °C, and the combination was acidified with 500 µL of 10% trichloroacetic acid (TCA). We combined 0.5 mL of supernatant with 100 µL of FeCl3 (0.1%) and 500 µL of distilled water. At 700 nm, the absorbance was measured. 

#### 2.4.3. Total Antioxidant Capacity (TAC)

At acidic pH, molybdate (VI) is reduced to molybdate (V), followed by the production of a green Mo (V)–phosphate complex. We added 2 mL of reagent solution (0.6 mol/L sulphuric acid, 28 mmol/L sodium phosphate, and 4 mmol/L ammonium molybdate) to 200 µL of a known concentration. 

After being incubated at 95 °C for 90 min, the mixtures were then cooled to room temperature (25 + 1 °C). To determine the absorbance, 695 nm was used. The overall antioxidant activity was evaluated as the amount of equivalent ascorbic acid per gram of extract (mg EAA/g extract) [30,31].

### 2.5. Antimicrobial Activity of S. elaeagnifolium Hydro-Ethanolic Extract and Hydro-Acetonic Extract

#### 2.5.1. Microbial Strains Used

The HEE and (HAE) of the plant studied were evaluated for antimicrobial activity against one fungal strain (*Candida albicans* ATCC 10231) and four bacterial strains (*Staphylococcus aureus* ATCC 6633, *Escherichia coli* K12, *Bacillus subtilis* DSM 6333, and *Proteus mirabilis* ATCC 29906), which have been provided by the Laboratory of Biotechnology, Environment, Agri-food, and Health, Faculty of Sciences Dhar El Mahraz, Sidi Mohammed Ben Abdellah University, Fez, Morocco.

#### 2.5.2. Antimicrobial Activity

The antibacterial efficacy of *S. elaeagnifolium* HEE and HAE was evaluated using the disk diffusion method [32]. Petri dishes containing MH (Mueller–Hinton) medium were inoculated with the four bacterial strains and *C. albicans* with the double-layer method. From the freshly grown cultures in MH medium, decimal dilutions were made in sterile physiological serum (0.9%) to reach a turbidity of 0.5 McFarland (10^8^ CFU/mL), of which 100 µL was added to tubes containing 5 mL of soft agar (0.5% agar), and then the inoculated tubes were spread in Petri dishes containing MH medium. Sterile Whatman discs (6 mm diameter) were placed in the centre of the Petri dish and then impregnated with 20 μL of *S. elaeagnifolium* HEE and HAE at a concentration of 30 mg/mL in 10% DMSO. The inoculated Petri plates were incubated at 37 °C, and the inhibition diameters for the four bacterial strains were examined after 24 h and for *C. albicans* after 48 h [32,33,34].

#### 2.5.3. Inhibitory Minimum Concentration (MIC)

To determine the minimum inhibitory concentration of *S. elaeagnifolium* HEE and HAE against *C. albicans* ATCC 10231 and the four bacterial strains, the microdilution method was used [33]. The sterile 96-well microplates were labelled, and the preparation of the microplates was done in an aseptic manner. Next, 100 μL of *S. elaeagnifolium* HEE and HAE in 10% (*v/v*) DMSO were pipetted into the plate’s first row. 50 μL of sterile MH was injected to each of the other wells. Using a multichannel pipette, serial dilutions were performed, and then 40 μL of each strain’s microbial solution was added to each well. Following a 48-hour incubation period for *C. albicans* and 24 h for the pathogenic bacteria at 37 °C [30,33], The colourimetric method (TTC 0.2 percent (*w/v*)) was used to estimate the MIC [35].

### 2.6. Statistical Analysis

Utilizing GraphPad Prism 8.0.1 and Minitab 19.1 software from GraphPad Software Inc. in San Diego, California, the data were statistically analyzed. The data were presented as means of three replicate experiments SD (standard deviation), and analysis of variance was used to examine whether there was a significant difference between the means (one-way ANOVA, Tukey’s test at *p* 0.05).

## 3. Results and Discussion

### 3.1. Yield of Extracts

The extraction of *S. elaeagnifolium* leaves performed by maceration gave yields of 10.2% for the HEE and 9.36% for the HAE. The extraction yield of *S. elaeagnifolium* depended on the extraction solvents and the plant part. Feki et al. [36] collected the S. *elaeagnifolium* plant (for seed study) in December 2011 (final maturity stage) in Monastir. They found the methanol extract of *S. elaeagnifolium* seeds presented the highest yields at 7.7%, followed by the acetone extract at 5.31%.

### 3.2. Phytochemical Study

#### 3.2.1. Phytochemical Screening

The preliminary tests consisted of qualitative characterisation processes to detect the various families of chemicals present in the leaves of *S. elaeagnifolium*. Specific reagents caused precipitation or colouring in these reactions. Table 1 shows the results of this phytochemical screening. It reveals if a group of secondary metabolites was present or absent.

Research on this plant has been very limited. It has been shown that the chemical composition of *S. elaeagnifolium* depends on the extraction solvent. Rajalakshmi and Pugalenthi (2016) performed a phytochemical screening for ethanol and petroleum ether extracts of *Solanum elaeagnifolium* leaves. The screening’s findings demonstrated that tannins, flavonoids, steroids, alkaloids, and saponins were all detected in the ethanol extract. The petroleum ether extract, however, only demonstrated the existence of steroids [37].

#### 3.2.2. Total Polyphenols and Flavonoids

In Table 2, the two extracts of S. elaeagnifolium leaves made using various extraction solvents are compared for their total phenol and flavonoid contents.

With the use of the Folin-Ciocalteu reagent and aluminum trichloride, respectively, the assessment of the amounts of polyphenols and flavonoids in the various extracts is assessed. From the different concentrations of gallic acid, the regression equation, y = 3.7142x − 0.0913, R² = 0.9950, for the polyphenol content gave 1.58 ± 0.03 mg EAG/g of hydro-acetonic and 2.54 ± 04 mg EAG/g of hydro-ethanolic extract. The calibration curve for quercetin and the regression equation, y = 11.786 + 0.0990, R² = 0.991, show that the flavonoid concentration equivalent to the quercetin concentrations of the hydro-acetonic extract was equal to 0.067 ± 0.001 mg EQ/g, while the hydro-ethanolic resulted in 0.012 ± 0.001 mg EQ/g extract. The amount of total phenol and flavonoid content varies according on the plant part used [38]. In comparison with the result of another study carried out in Tunisia, where they were worked on the fruits of *S. elaeagnifolium* at different stages of ripening and with different solvents, the flavonoid contents of ethyl acetate extracts (61.09 ± 1.92 mg EQ/g extract), dichloromethane (34.17 ± 0.22 mg EQ/g extract), and methanol (17.62 ± 0.42 mg EQ/g extract) were determined for ripe fruits in this study. Unripe fruit has a total polyphenol content of 2.29 ± 12.79 mg/g extract, while ripe fruit has a total polyphenol value of 1.92 ± 9.04 mg/g extract [14].

### 3.3. Antioxidant Activity

#### 3.3.1. DPPH Free Radical Scavenging Activity

**Figure 2:**
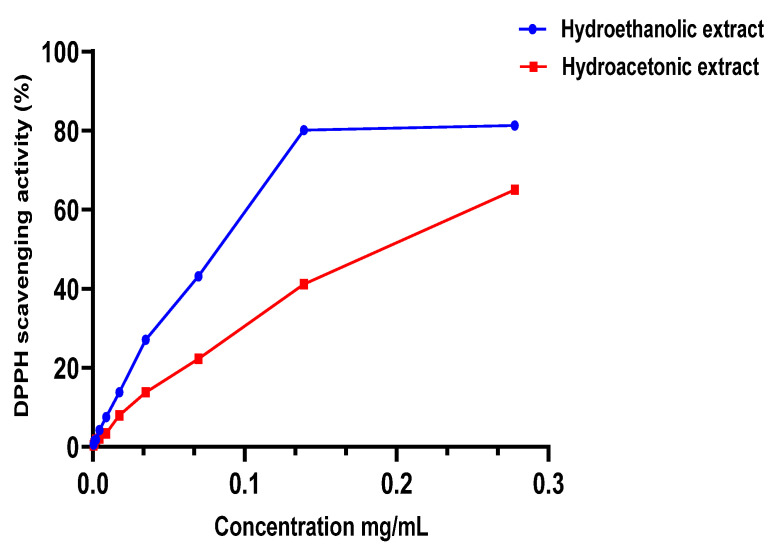
DPPH scavenging activity of extracts.

**Figure 3:**
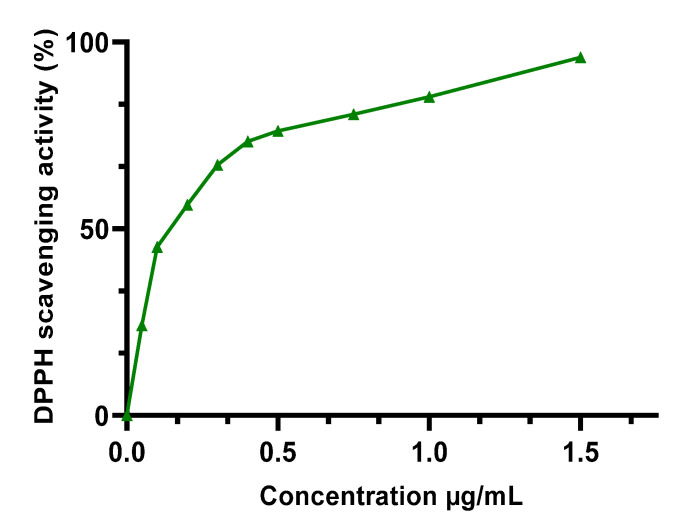
DPPH scavenging activity of BHT.

The m edian inhibitory concentration (IC50) of the extracts studied (Table 3) was calculated using the Figure 2 and Figure 3 after measuring the absorbance. HEE showed excellent antioxidant capacity, higher than HAE, with an IC50 of 0.0807 ± 0.0039 mg/mL, while HAE had an IC50 of 0.198 ± 0.0196 mg/mL. Both extracts have less antioxidant capacity than the BHT standard, which had an IC50 of 0.122 ± 0.021 μg/mL. These findings are in accordance with the polyphenol content of the two extracts (HEE > HAE); so, these chemicals can be used to explain the antioxidant capacity observed in the DPPH test. To verify the existence of these compounds, we used a DPPH assay. This relationship has already been established, and other writers have reported it using similar testing techniques [21,39].

#### 3.3.2. Reductive Power Test (FRAP)

The FRAP test served as a further evaluation of the reducing power of *S. elaeagnifolium* extracts. Antioxidant compounds that break free radical chains through hydrogen atom donation are typically associated with reducing power [40]. The HAE exhibited greater antioxidant activity (EC50 = 0.08256 ± 0.005105 mg/mL) than the HEE (EC50 = 0.1157 ± 0.0400 mg/mL), but this activity remained lower than BHT (0.362 ± 0.010 µg/mL).

The variation in chemical composition and polyphenol and flavonoid concentration between both the two extracts can be used to justify the difference in the ability to reduce iron between the two extracts. It’s conceivable that the extraction agent caused this result [24].

#### 3.3.3. Total Antioxidant Capacity (TAC)

**Figure 4:**
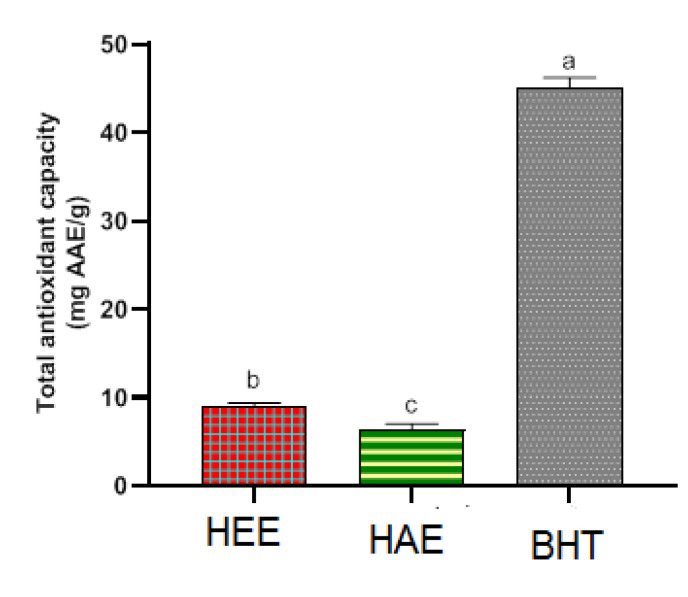
Antioxidant capacity overall of extracts. Means with distinct letters differ from one another significantly (*p* < 0.05).

Antioxidant activity was discovered in both *S. elaeagnifolium* leaf extracts (Figure 4). The hydro-ethanolic extract showed the highest total antioxidant activity 8.95 ± 0.42 mg AAE/g, The hydro-acetonic extract was the next, with a total antioxidant activity of 6.44 ± 0.61 mg AAE/g. Statistical significance (p < 0.05) was seen in the TAC difference between the two *S. elaeagnifolium* extracts. Rajalakshmi and Pugalenthi (2016) have worked on the whole plant of *S. elaeagnifolium*. Using organic solvent extraction, ethanol generally showed better TAC. Maximum total antioxidant activity was presented by ethanol extract of *S. elaeagnifolium* (19.4 mg/mL), followed by petroleum ether extract of *S. elaeagnifolium* (1.5 mg/mL) [37]. The extraction solvent can influence the chemical composition of the extract; this was reflected in the difference of the total antioxidant capacity [41]. According to the findings of Feki et al. (2014), There is a strong and linear correlation between antioxidant activity and *S. elaeagnifolium*’s phenolic content, indicating that phenolic chemicals may be very important in antioxidant activity. [36].

### 3.4. Antimicrobial Activity of S. elaeagnifolium Hydro-Ethanolic Extract and Hydro-Acetonic Extract

**Figure 5:**
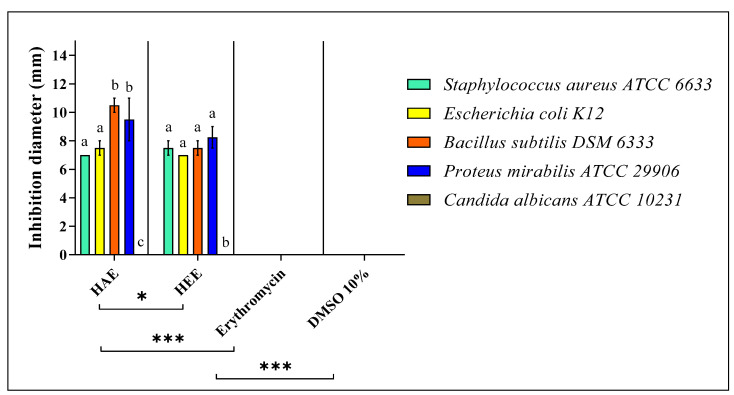
Antimicrobial activity of *S. elaeagnifolium* HEE and HAE in comparison with the antibiotic Erythromycin. Means (± SD, n = 3) denoted by the same letter indicate no significant difference according to Tukey’s multiple range tests at *p* < 0.05. The signs * and *** mean that there is a significant difference between the different samples and controls.

**Figure 6:**
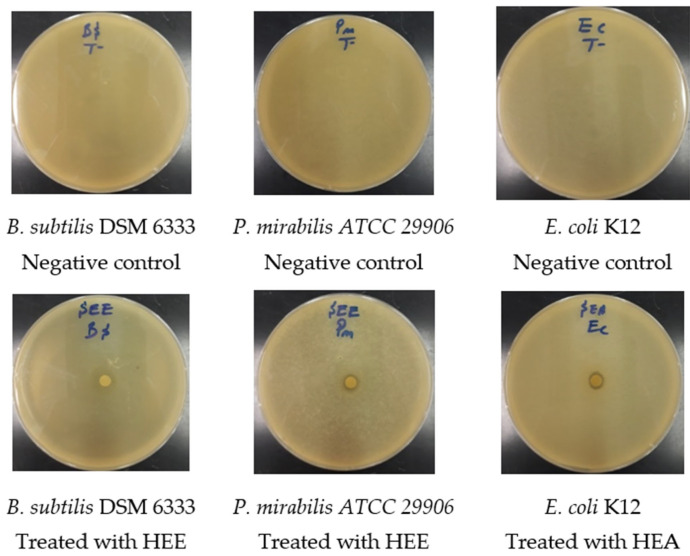
Photographs showing the effects of extracts on some of the bacteria tested.

The results of the antimicrobial activity test and the MIC of *S. elaeagnifolium* HEE and HAE against the four bacterial strains and *C. albicans* ATCC 10231 are summarised in (Figure 5 and Figure 6) and (Table 4), respectively. The difference in the inhibition diameters could be mainly due to the chemical composition of *S. elaeagnifolium* HAE and HEE, and the antibacterial activity of the latter could be mainly due to the compounds in these two extracts. Both HAE and HEE of *S. elaeagnifolium* exhibit interesting antibacterial activity compared to the antibiotic erythromycin. HAE shows the highest inhibitory activity against *Bacillus subtilis* DSM 6333 with an inhibition diameter of 10.5 ± 0.50 mm and MIC of 7.5 ± 0.00 mg/mL. HEE shows the highest activity against *Proteus mirabilis* ATCC 29906 with an inhibition diameter of 8.25 ± 0.75 mm and MIC of 15 ± 0.00 mg/mL. Between the two extracts, the HAE was found to be more potent than HEE. Furthermore, both extracts were shown to be bactericidal against the four bacterial strains tested. However, *S. elaeagnifolium* HAE and HEE did not show any activity against *C. albicans* strain ATCC 10231. Scientific studies focusing on the activity of *S. elaeagnifolium* extracts are very rare. To our knowledge, no detailed information is available on the antimicrobial activities, especially the antifungal properties, of *S. elaeagnifolium* in the literature. Our findings are consistent with those of [23], which indicated that HAE and HEE of *S. elaeagnifolium* Cav. show significant antibacterial activity against some selected human pathogenic bacteria, such as *Escherichia coli, Staphylococcus aureus, Klebsiella oxytoca, Bacillus cereus, Serratia marcescens, Klebsiella pneumonia, Enterobacter amnigenus, Staphylococcus lentus, Staphylococcus haemolyticus,* and *Brevibacterium paucivorans*, and also with the study of [42], which demonstrates that *S. elaeagnifolium* extract does not show any activity against *C. albicans*, but our results are in contradiction with the same study for *S. aureus* results. The promising antibacterial activity of HAE and HEE against pathogenic bacterial strains may be mainly due to the chemical composition of *S. elaeagnifolium*, especially the presence of flavonoids, polyphenols, alkaloids, and saponins. Several research papers have been reported to indicate that these chemical compounds exhibit significant antimicrobial activities. Saponins are naturally occurring compounds that possess a multitude of biological activities, especially antibacterial and antifungal activity [43]. The study by [44] showed that flavonoids and saponins exhibit antibacterial properties due to their ability to form complexes with soluble proteins, extracellular proteins, and the bacterial cell wall. Also, Ref. [45] showed that flavonoids have significant antifungal properties against Candida spp. As polyphenols have powerful antibacterial properties, they could be crucial in a new approach to treating pathogenic microbes [46,47]. Alkaloids as well exhibit significant antibacterial activities against pathogenic microbial strains [48]. As we have previously described, both HAE and HEE of *S. elaeagnifolium* exhibit significant antibacterial activities against antibiotic-resistant human pathogenic bacteria, suggesting the use of the bioactive molecules contained in these extracts as an alternative to commercially available antibiotics to combat microbial resistance.

### 3.5. Relationships between Studied Parameters of S. elaeagnifolium

**Figure 7:**
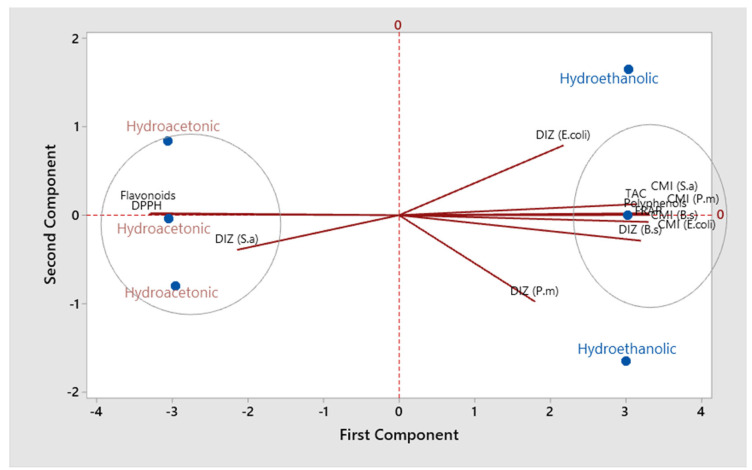
Factor analysis (PCA) of the examined extracts using the evaluated parameters.

Table 5 shows the correlation coefficients between the different studied parameters on the two *S. elaeagnifolium* extracts. Polyphenols were negatively correlated with Flavonoids and DPPH IC50 (r =−0.99; *p* < 0.001), while they were positively correlated with FRAP (r = 0.99; *p* < 0.001), TAC (r = 0.94; *p* < 0.01), DIZ (B.s) (r = 0.96; *p* < 0.01), and MICs (r = 0.99; *p* < 0.001) against all strains. On the other hand, flavonoids have a positive correlation with DPPH and a negative correlation with the other parameters. Figure 7 displays the findings of the main components analysis (PCA). The eigenvalues of the first two principal components (PC) are greater than 1. These two PC represent 94.7% of the variation in the data. The initial primary component PC1 (84.3%) shows a positive association with polyphenols, FRAP, and the MICs of all studied strains, while it has a negative association with flavonoids and DPPH. The second component PC2 (10.4%) shows a strong negative association with DIZ (P*. m*) and a positive association with DIZ (E. *coli*). The projected scoring diagram and contribution diagram show the contrast between the different extracts in a very apparent way. The HEE is characterised by the high content of polyphenols and their effect on antibacterial and antioxidant activities. While the HAE is characterised by the flavonoids compounds, the low concentration of bacteriostatic and antioxidant activities (FRAP). Indeed, the negative correlation between polyphenols and DPPH and between flavonoids and FRAP has been described in a number of academic papers. Depending on the characteristics of each molecule and the amount present in the raw material, flavonoids and other polyphenols have different antioxidant capacities [49,50,51,52,53]. The MIC of all studied strains showed a strong negative correlation with flavonoid compounds. From these results, it can be indicated that the presence of flavonoids in the plant extract contributed to the lethality of the studied strains at low concentrations. Indeed, according to several studies, antibacterial activity is strongly correlated with the phenolic compounds [54].

## 4. Conclusions

In conclusion, the antioxidant and antibacterial activity of the hydro-ethanolic and hydro-acetonic extract of the leaves of *S. elaeagnifolium* grown in Morocco were investigated as well as phytochemical screening. The findings of this study demonstrate that both extracts have antioxidant activity while also having antibacterial activity against bacteria and yeasts using the standard utilised. The two extracts were shown to have a moderate amount of polyphenols and flavonoids. These activities are explained by the plant’s richness in phenols, flavonoids, alkaloids, and tannins. These results encourage us to study other biological activities of this plant. Thus, after conducting some advanced studies, the phytochemicals could be separated and the biosynthesis and structural elucidations could be carried out in the future for the effective utilization of these potentially medicinal plants. Thus, HPLC or GC/MS could be carried out in order to know the main molecules of *S. elaeagnifolium.*

## Figures and Tables

**Table 1:** Secondary metabolites presented in the studied extract.

Tests	Solvent Extracts	
	Hydro-Ethanolic Extract (HEE)	Hydro-Acetonic Extract (HAE)
Tannins	+	+
Flavonoids	+	+
Alkaloids	+	+
Polyphenols	+	+
Saponins	+	−
Steroids	+	+

+: Presence; −: Absent.

**Table 2:** Measurement of phytochemical compound concentration.

Extract	Polyphenols (mg EAG/g Extract)	Flavonoids (mg EQ/g Extract)
Hydro-ethanolic	2.54 ± 0.4 ^a^	0.012 ± 0.001 ^a^
Hydro-acetonic	1.58 ± 0.03 ^b^	0.067 ± 0.001 ^b^

Values in each column that have a distinct letter are statistically different (*p* < 0.05).

**Table 3:** DPPH-IC50 and FRAP-EC50 of hydro-ethanolic and hydro-acetonic extracts of *S. elaeagnifolium* compared with BHT.

	Hydro-Ethanolic	Hydro-Acetonic	BHT
DPPH-IC50	0.0807 ± 0.0039 mg/mL ^b^	0.198 ± 0.0196 mg/mL ^c^	0.122 ± 0.0210 μg/mL ^a^
FRAP-EC50	0.0825 ± 0.0051 mg/mL ^b^	0.1157 ± 0.0400 mg/mL ^c^	0.362 ± 0.0100 µg/mL ^a^

There are significant differences between the mean values (SD, *n* = 3) that are followed by various letters in the same row (one-way ANOVA; Tukey’s test, *p <* 0.05).

**Table 4:** MIC of antimicrobial activity of *S. elaeagnifolium* HEE and HAE.

	S. aureus ATCC 6633	E. coli K12	B. subtilis DSM 6333	P. mirabilis ATCC 29906	C. albicans ATCC 10231
*S. elaeagnifolium* HEE (mg/mL)	7.5 ± 0.00 ^a^	7.5 ± 0.00 ^a^	15 ± 0.00 ^b^	15 ± 0.00 ^b^	-
*S. elaeagnifolium* HAE (mg/mL)	3.75 ± 0.00 ^a^	3.75 ± 0.00 ^a^	7.5 ± 0.00 ^b^	7.5 ± 0.00 ^b^	-

Mean values (± SD, *n* = 3) followed by different letters in same row are significantly different (One-way ANOVA; Tukey’s test*, p* < 0.05).

**Table 5:** Correlation between studied parameters of *S. elaeagnifolium*.

	Polyphenols	Flavonoids	DPPH	FRAP	TAC	DIZ (S.a)	DIZ (E. coli)	DIZ (B.s)	DIZ (P.m)	CMI (S.a)	CMI (E. coli)	CMI (B.s)	CMI (P.m)
Polyphenols	1.000												
Flavonoids	−0.999	1.000											
DPPH	−0.995	0.994	1.000										
FRAP	0.992	−0.997	−0.991	1.000									
TAC	0.943	−0.946	−0.924	0.932	1.000								
DIZ (S.a)	−0.646	0.646	0.613	−0.645	−0.513	1.000							
DIZ (E. coli)	0.673	−0.650	−0.652	0.600	0.759	−0.429	1.000						
DIZ (B.s)	0.960	−0.968	−0.966	0.977	0.906	−0.491	0.491	1.000					
DIZ (P.m)	0.526	−0.551	−0.554	0.597	0.429	−0.071	−0.213	0.733	1.000				
MIC (S.a)	0.998	−0.999	−0.993	0.997	0.947	−0.655	0.655	0.965	0.542	1.000			
MIC (E. coli)	0.998	−0.999	−0.993	0.997	0.947	−0.655	0.655	0.965	0.542	1.000	1.000		
MIC (B.s)	0.998	−0.999	−0.993	0.997	0.947	−0.655	0.655	0.965	0.542	1.000	1.000	1.000	
MIC (P.m)	0.998	−0.999	−0.993	0.997	0.947	−0.655	0.655	0.965	0.542	1.000	1.000	1.000	1.000

## Data Availability

Not applicable.

## Abbreviations

HEE	Hydro-ethanolic extract
HAE	Hydro-acetonic extract
MIC	Minimum inhibitory concentration
DPPH	2,2’-diphenyl-1 picrylhydrazyl
TAC	Total antioxidant capacity
TPC	Total polyphenol content
TFC	Total flavonoids content
